# Tracheal intubation in traumatic brain injury: a multicentre prospective observational study

**DOI:** 10.1016/j.bja.2020.05.067

**Published:** 2020-07-31

**Authors:** Benjamin Yael Gravesteijn, Charlie Aletta Sewalt, Daan Nieboer, David Krishna Menon, Andrew Maas, Fiona Lecky, Markus Klimek, Hester Floor Lingsma, CENTER-TBI collaborators, Cecilia Åkerlund, Krisztina Amrein, Nada Andelic, Lasse Andreassen, Audny Anke, Anna Antoni, Gérard Audibert, Philippe Azouvi, Maria Luisa Azzolini, Ronald Bartels, Pál Barzó, Romuald Beauvais, Ronny Beer, Bo-Michael Bellander, Antonio Belli, Habib Benali, Maurizio Berardino, Luigi Beretta, Morten Blaabjerg, Peter Bragge, Alexandra Brazinova, Vibeke Brinck, Joanne Brooker, Camilla Brorsson, Andras Buki, Monika Bullinger, Manuel Cabeleira, Alessio Caccioppola, Emiliana Calappi, Maria Rosa Calvi, Peter Cameron, Guillermo Carbayo Lozano, Marco Carbonara, Simona Cavallo, Giorgio Chevallard, Arturo Chieregato, Giuseppe Citerio, Iris Ceyisakar, Hans Clusmann, Mark Coburn, Jonathan Coles, Jamie D. Cooper, Marta Correia, Amra Čović, Nicola Curry, Endre Czeiter, Marek Czosnyka, Claire Dahyot-Fizelier, Paul Dark, Helen Dawes, Véronique De Keyser, Vincent Degos, Francesco Della Corte, Hugo den Boogert, Bart Depreitere, Đula Đilvesi, Abhishek Dixit, Emma Donoghue, Jens Dreier, Guy-Loup Dulière, Ari Ercole, Patrick Esser, Erzsébet Ezer, Martin Fabricius, Valery L. Feigin, Kelly Foks, Shirin Frisvold, Alex Furmanov, Pablo Gagliardo, Damien Galanaud, Dashiell Gantner, Guoyi Gao, Pradeep George, Alexandre Ghuysen, Lelde Giga, Ben Glocker, Jagoš Golubovic, Pedro A. Gomez, Johannes Gratz, Benjamin Gravesteijn, Francesca Grossi, Russell L. Gruen, Deepak Gupta, Juanita A. Haagsma, Iain Haitsma, Raimund Helbok, Eirik Helseth, Lindsay Horton, Jilske Huijben, Peter J. Hutchinson, Bram Jacobs, Stefan Jankowski, Mike Jarrett, Ji-yao Jiang, Faye Johnson, Kelly Jones, Mladen Karan, Angelos G. Kolias, Erwin Kompanje, Daniel Kondziella, Evgenios Koraropoulos, Lars-Owe Koskinen, Noémi Kovács, Ana Kowark, Alfonso Lagares, Linda Lanyon, Steven Laureys, Fiona Lecky, Didier Ledoux, Rolf Lefering, Valerie Legrand, Aurelie Lejeune, Leon Levi, Roger Lightfoot, Hester Lingsma, Andrew I.R. Maas, Ana M. Castaño-León, Marc Maegele, Marek Majdan, Alex Manara, Geoffrey Manley, Costanza Martino, Hugues Maréchal, Julia Mattern, Catherine McMahon, Béla Melegh, David Menon, Tomas Menovsky, Ana Mikolic, Benoit Misset, Visakh Muraleedharan, Lynnette Murray, Ancuta Negru, David Nelson, Virginia Newcombe, Daan Nieboer, József Nyirádi, Otesile Olubukola, Matej Oresic, Fabrizio Ortolano, Aarno Palotie, Paul M. Parizel, Jean-François Payen, Natascha Perera, Vincent Perlbarg, Paolo Persona, Wilco Peul, Anna Piippo-Karjalainen, Matti Pirinen, Horia Ples, Suzanne Polinder, Inigo Pomposo, Jussi P. Posti, Louis Puybasset, Andreea Radoi, Arminas Ragauskas, Rahul Raj, Malinka Rambadagalla, Jonathan Rhodes, Sylvia Richardson, Sophie Richter, Samuli Ripatti, Saulius Rocka, Cecilie Roe, Olav Roise, Jonathan Rosand, Jeffrey V. Rosenfeld, Christina Rosenlund, Guy Rosenthal, Rolf Rossaint, Sandra Rossi, Daniel Rueckert, Martin Rusnák, Juan Sahuquillo, Oliver Sakowitz, Renan Sanchez-Porras, Janos Sandor, Nadine Schäfer, Silke Schmidt, Herbert Schoechl, Guus Schoonman, Rico Frederik Schou, Elisabeth Schwendenwein, Charlie Sewalt, Toril Skandsen, Peter Smielewski, Abayomi Sorinola, Emmanuel Stamatakis, Simon Stanworth, Robert Stevens, William Stewart, Ewout W. Steyerberg, Nino Stocchetti, Nina Sundström, Anneliese Synnot, Riikka Takala, Viktória Tamás, Tomas Tamosuitis, Mark Steven Taylor, Braden Te Ao, Olli Tenovuo, Alice Theadom, Matt Thomas, Dick Tibboel, Marjolein Timmers, Christos Tolias, Tony Trapani, Cristina Maria Tudora, Peter Vajkoczy, Shirley Vallance, Egils Valeinis, Zoltán Vámos, Mathieu van der Jagt, Gregory Van der Steen, Joukje van der Naalt, Jeroen T.J.M. van Dijck, Thomas A. van Essen, Wim Van Hecke, Caroline van Heugten, Dominique Van Praag, Thijs Vande Vyvere, Roel P.J. van Wijk, Alessia Vargiolu, Emmanuel Vega, Kimberley Velt, Jan Verheyden, Paul M. Vespa, Anne Vik, Rimantas Vilcinis, Victor Volovici, Nicole von Steinbüchel, Daphne Voormolen, Petar Vulekovic, Kevin K.W. Wang, Eveline Wiegers, Guy Williams, Lindsay Wilson, Stefan Winzeck, Stefan Wolf, Zhihui Yang, Peter Ylén, Alexander Younsi, Frederick A. Zeiler, Veronika Zelinkova, Agate Ziverte, Tommaso Zoerle

**Affiliations:** 6 Department of Physiology and Pharmacology, Section of Perioperative Medicine and Intensive Care, Karolinska Institutet, Stockholm, Sweden; 7 János Szentágothai Research Centre, University of Pécs, Pécs, Hungary; 8 Division of Surgery and Clinical Neuroscience, Department of Physical Medicine and Rehabilitation, Oslo University Hospital and University of Oslo, Oslo, Norway; 9 Department of Neurosurgery, University Hospital Northern Norway, Tromso, Norway; 10 Department of Physical Medicine and Rehabilitation, University Hospital Northern Norway, Tromso, Norway; 11 Trauma Surgery, Medical University Vienna, Vienna, Austria; 12 Department of Anesthesiology & Intensive Care, University Hospital Nancy, Nancy, France; 13 Raymond Poincare Hospital, Assistance Publique – Hopitaux de Paris, Paris, France; 14 Department of Anesthesiology & Intensive Care, S. Raffaele University Hospital, Milan, Italy; 15 Department of Neurosurgery, Radboud University Medical Center, Nijmegen, Netherlands; 16 Department of Neurosurgery, University of Szeged, Szeged, Hungary; 17 International Projects Management, ARTTIC, Munich, Germany; 18 Department of Neurology, Neurological Intensive Care Unit, Medical University of Innsbruck, Innsbruck, Austria; 19 Department of Neurosurgery & Anesthesia & Intensive Care Medicine, Karolinska University Hospital, Stockholm, Sweden; 20 NIHR Surgical Reconstruction and Microbiology Research Centre, Birmingham, UK; 21 Anesthesie-Réanimation, Assistance Publique – Hopitaux de Paris, Paris, France; 22 Department of Anesthesia & ICU, AOU Città della Salute e della Scienza di Torino – Orthopedic and Trauma Center, Turin, Italy; 23 Department of Neurology, Odense University Hospital, Odense, Denmark; 24 BehaviourWorks, Monash Sustainability Institute, Monash University, Victoria, Australia; 25 Department of Public Health, Faculty of Health Sciences and Social Work, Trnava University, Trnava, Slovakia; 26 Quesgen Systems Inc., Burlingame, CA, USA; 27 Australian & New Zealand Intensive Care Research Centre, Department of Epidemiology and Preventive Medicine, School of Public Health and Preventive Medicine, Monash University, Melbourne, Australia; 28 Department of Surgery and Perioperative Science, Umeå University, Umeå, Sweden; 29 Department of Neurosurgery, Medical School, University of Pécs, Hungary and Neurotrauma Research Group, János Szentágothai Research Centre, University of Pécs, Pécs, Hungary; 30 Department of Medical Psychology, Universitätsklinikum Hamburg-Eppendorf, Hamburg, Germany; 31 Brain Physics Lab, Division of Neurosurgery, Dept of Clinical Neurosciences, University of Cambridge, Addenbrooke’s Hospital, Cambridge, UK; 32 Neuro ICU, Fondazione IRCCS Cà Granda Ospedale Maggiore Policlinico, Milan, Italy; 33 ANZIC Research Centre, Monash University, Department of Epidemiology and Preventive Medicine, Melbourne, Victoria, Australia; 34 Department of Neurosurgery, Hospital of Cruces, Bilbao, Spain; 35 NeuroIntensive Care, Niguarda Hospital, Milan, Italy; 36 School of Medicine and Surgery, Università Milano Bicocca, Milano, Italy; 37 NeuroIntensive Care, ASST di Monza, Monza, Italy; 38 Department of Public Health, Erasmus Medical Center-University Medical Center, Rotterdam, Netherlands; 39 Department of Neurosurgery, Medical Faculty RWTH Aachen University, Aachen, Germany; 40 Department of Anaesthesiology, University Hospital of Aachen, Aachen, Germany; 41 Department of Anesthesia & Neurointensive Care, Cambridge University Hospital NHS Foundation Trust, Cambridge, UK; 42 School of Public Health & PM, Monash University and The Alfred Hospital, Melbourne, Victoria, Australia; 43 Radiology/MRI department, MRC Cognition and Brain Sciences Unit, Cambridge, UK; 44 Institute of Medical Psychology and Medical Sociology, Universitätsmedizin Göttingen, Göttingen, Germany; 45 Oxford University Hospitals NHS Trust, Oxford, UK; 46 Intensive Care Unit, CHU Poitiers, Potiers, France; 47 University of Manchester NIHR Biomedical Research Centre, Critical Care Directorate, Salford Royal Hospital NHS Foundation Trust, Salford, UK; 48 Movement Science Group, Faculty of Health and Life Sciences, Oxford Brookes University, Oxford, UK; 49 Department of Neurosurgery, Antwerp University Hospital and University of Antwerp, Edegem, Belgium; 50 Department of Anesthesia & Intensive Care, Maggiore Della Carità Hospital, Novara, Italy; 51 Department of Neurosurgery, University Hospitals Leuven, Leuven, Belgium; 52 Department of Neurosurgery, Clinical centre of Vojvodina, Faculty of Medicine, University of Novi Sad, Novi Sad, Serbia; 53 Division of Anaesthesia, University of Cambridge, Addenbrooke’s Hospital, Cambridge, UK; 54 Center for Stroke Research Berlin, Charité – Universitätsmedizin Berlin, corporate member of Freie Universität Berlin, Humboldt-Universität zu Berlin, and Berlin Institute of Health, Berlin, Germany; 55 Intensive Care Unit, CHR Citadelle, Liège, Belgium; 56 Department of Anaesthesiology and Intensive Therapy, University of Pécs, Pécs, Hungary; 57 Departments of Neurology, Clinical Neurophysiology and Neuroanesthesiology, Region Hovedstaden Rigshospitalet, Copenhagen, Denmark; 58 National Institute for Stroke and Applied Neurosciences, Faculty of Health and Environmental Studies, Auckland University of Technology, Auckland, New Zealand; 59 Department of Neurology, Erasmus MC, Rotterdam, Netherlands; 60 Department of Anesthesiology and Intensive care, University Hospital Northern Norway, Tromso, Norway; 61 Department of Neurosurgery, Hadassah-hebrew University Medical center, Jerusalem, Israel; 62 Fundación Instituto Valenciano de Neurorrehabilitación (FIVAN), Valencia, Spain; 63 Department of Neurosurgery, Shanghai Renji hospital, Shanghai Jiaotong University/School of Medicine, Shanghai, China; 64 Karolinska Institutet, INCF International Neuroinformatics Coordinating Facility, Stockholm, Sweden; 65 Emergency Department, CHU, Liège, Belgium; 66 Neurosurgery clinic, Pauls Stradins Clinical University Hospital, Riga, Latvia; 67 Department of Computing, Imperial College London, London, UK; 68 Department of Neurosurgery, Hospital Universitario 12 de Octubre, Madrid, Spain; 69 Department of Anesthesia, Critical Care and Pain Medicine, Medical University of Vienna, Vienna, Austria; 70 College of Health and Medicine, Australian National University, Canberra, Australia; 71 Department of Neurosurgery, Neurosciences Centre & JPN Apex Trauma Centre, All India Institute of Medical Sciences, New Delhi, India; 72 Department of Neurosurgery, Erasmus MC, Rotterdam, Netherlands; 73 Department of Neurosurgery, Oslo University Hospital, Oslo, Norway; 74 Division of Psychology, University of Stirling, Stirling, UK; 75 Division of Neurosurgery, Department of Clinical Neurosciences, Addenbrooke’s Hospital & University of Cambridge, Cambridge, UK; 76 Department of Neurology, University of Groningen, University Medical Center Groningen, Groningen, Netherlands; 77 Neurointensive Care, Sheffield Teaching Hospitals NHS Foundation Trust, Sheffield, UK; 78 Salford Royal Hospital NHS Foundation Trust Acute Research Delivery Team, Salford, UK; 79 Department of Intensive Care and Department of Ethics and Philosophy of Medicine, Erasmus Medical Center, Rotterdam, Netherlands; 80 Department of Clinical Neuroscience, Neurosurgery, Umeå University, Umeå, Sweden; 81 Hungarian Brain Research Program – Grant No. KTIA_13_NAP-A-II/8, University of Pécs, Pécs, Hungary; 82 Cyclotron Research Center, University of Liège, Liège, Belgium; 83 Centre for Urgent and Emergency Care Research (CURE), Health Services Research Section, School of Health and Related Research (ScHARR), University of Sheffield, Sheffield, UK; 84 Emergency Department, Salford Royal Hospital, Salford, UK; 85 Institute of Research in Operative Medicine (IFOM), Witten/Herdecke University, Cologne, Germany; 86 VP Global Project Management CNS, ICON, Paris, France; 87 Department of Anesthesiology-Intensive Care, Lille University Hospital, Lille, France; 88 Department of Neurosurgery, Rambam Medical Center, Haifa, Israel; 89 Department of Anesthesiology & Intensive Care, University Hospitals Southhampton NHS Trust, Southhampton, UK; 90 Cologne-Merheim Medical Center (CMMC), Department of Traumatology, Orthopedic Surgery and Sportmedicine, Witten/Herdecke University, Cologne, Germany; 91 Intensive Care Unit, Southmead Hospital, Bristol, UK; 92 Department of Neurological Surgery, University of California, San Francisco, CA, USA; 93 Department of Anesthesia & Intensive Care, M. Bufalini Hospital, Cesena, Italy; 94 Department of Neurosurgery, University Hospital Heidelberg, Heidelberg, Germany; 95 Department of Neurosurgery, The Walton centre NHS Foundation Trust, Liverpool, UK; 96 Department of Medical Genetics, University of Pécs, Pécs, Hungary; 97 Department of Neurosurgery, Emergency County Hospital Timisoara, Timisoara, Romania; 98 School of Medical Sciences, Örebro University, Örebro, Sweden; 99 Institute for Molecular Medicine Finland, University of Helsinki, Helsinki, Finland; 100 Analytic and Translational Genetics Unit, Department of Medicine, Psychiatric & Neurodevelopmental Genetics Unit, Department of Psychiatry, Department of Neurology, Massachusetts General Hospital, Boston, MA, USA; 101 Program in Medical and Population Genetics, The Stanley Center for Psychiatric Research, The Broad Institute of MIT and Harvard, Cambridge, MA, USA; 102 Department of Radiology, University of Antwerp, Edegem, Belgium; 103 Department of Anesthesiology & Intensive Care, University Hospital of Grenoble, Grenoble, France; 104 Department of Anesthesia & Intensive Care, Azienda Ospedaliera Università di Padova, Padova, Italy; 105 Department of Neurosurgery, Leiden University Medical Center, Department of Neurosurgery, Medical Center Haaglanden, Leiden, The Hague, Netherlands; 106 Department of Neurosurgery, Helsinki University Central Hospital, Helsinki, Finland; 107 Division of Clinical Neurosciences, Department of Neurosurgery and Turku Brain Injury Centre, Turku University Hospital and University of Turku, Turku, Finland; 108 Department of Anesthesiology and Critical Care, Pitié -Salpêtrière Teaching Hospital, Assistance Publique, Hôpitaux de Paris and University Pierre et Marie Curie, Paris, France; 109 Neurotraumatology and Neurosurgery Research Unit (UNINN), Vall d'Hebron Research Institute, Barcelona, Spain; 110 Department of Neurosurgery, Kaunas University of technology and Vilnius University, Vilnius, Lithuania; 111 Department of Neurosurgery, Rezekne Hospital, Rezekne, Latvia; 112 Department of Anaesthesia, Critical Care & Pain Medicine NHS Lothian & University of Edinburg, Edinburgh, UK; 113 MRC Biostatistics Unit, Cambridge Institute of Public Health, Cambridge, UK; 114 Department of Physical Medicine and Rehabilitation, Oslo University Hospital/University of Oslo, Oslo, Norway; 115 Division of Orthopedics, Oslo University Hospital, Oslo, Norway; 116 Institue of Clinical Medicine, Faculty of Medicine, University of Oslo, Oslo, Norway; 117 Broad Institute, Cambridge MA Harvard Medical School, Massachusetts General Hospital, Boston, MA, USA; 118 National Trauma Research Institute, The Alfred Hospital, Monash University, Melbourne, Victoria, Australia; 119 Department of Neurosurgery, Odense University Hospital, Odense, Denmark; 120 International Neurotrauma Research Organisation, Vienna, Austria; 121 Klinik für Neurochirurgie, Klinikum Ludwigsburg, Ludwigsburg, Germany; 122 Division of Biostatistics and Epidemiology, Department of Preventive Medicine, University of Debrecen, Debrecen, Hungary; 123 Department Health and Prevention, University Greifswald, Greifswald, Germany; 124 Department of Anaesthesiology and Intensive Care, AUVA Trauma Hospital, Salzburg, Austria; 125 Department of Neurology, Elisabeth-TweeSteden Ziekenhuis, Tilburg, Netherlands; 126 Department of Neuroanesthesia and Neurointensive Care, Odense University Hospital, Odense, Denmark; 127 Department of Neuromedicine and Movement Science, Norwegian University of Science and Technology, NTNU, Trondheim, Norway; 128 Department of Physical Medicine and Rehabilitation, St. Olavs Hospital, Trondheim University Hospital, Trondheim, Norway; 129 Department of Neurosurgery, University of Pécs, Pécs, Hungary; 130 Division of Neuroscience Critical Care, John Hopkins University School of Medicine, Baltimore, MD, USA; 131 Department of Neuropathology, Queen Elizabeth University Hospital and University of Glasgow, Glasgow, UK; 132 Department of Department of Biomedical Data Sciences, Leiden University Medical Center, Leiden, Netherlands; 133 Department of Pathophysiology and Transplantation, Milan University, and Neuroscience ICU, Fondazione IRCCS Cà Granda Ospedale Maggiore Policlinico, Milan, Italy; 134 Department of Radiation Sciences, Biomedical Engineering, Umeå University, Umeå, Sweden; 135 Cochrane Consumers and Communication Review Group, Centre for Health Communication and Participation, School of Psychology and Public Health, La Trobe University, Melbourne, Australia; 136 Perioperative Services, Intensive Care Medicine and Pain Management, Turku University Hospital and University of Turku, Turku, Finland; 137 Department of Neurosurgery, Kaunas University of Health Sciences, Kaunas, Lithuania; 138 Intensive Care and Department of Pediatric Surgery, Erasmus Medical Center, Sophia Children’s Hospital, Rotterdam, Netherlands; 139 Department of Neurosurgery, Kings college London, London, UK; 140 Neurologie, Neurochirurgie und Psychiatrie, Charité – Universitätsmedizin Berlin, Berlin, Germany; 141 Department of Intensive Care Adults, Erasmus MC– University Medical Center Rotterdam, Rotterdam, Netherlands; 142 icoMetrix NV, Leuven, Belgium; 143 Movement Science Group, Faculty of Health and Life Sciences, Oxford Brookes University, Oxford, UK; 144 Psychology Department, Antwerp University Hospital, Edegem, Belgium; 145 Director of Neurocritical Care, University of California, Los Angeles, CA, USA; 146 Department of Neurosurgery, St. Olavs Hospital, Trondheim University Hospital, Trondheim, Norway; 147 Department of Emergency Medicine, University of Florida, Gainesville, FL, USA; 148 Department of Neurosurgery, Charité – Universitätsmedizin Berlin, corporate member of Freie Universität Berlin, Humboldt-Universität zu Berlin, and Berlin Institute of Health, Berlin, Germany; 149 VTT Technical Research Centre, Tampere, Finland; 150 Section of Neurosurgery, Department of Surgery, Rady Faculty of Health Sciences, University of Manitoba, Winnipeg, MB, Canada; 1 Department of Anaesthesiology, Erasmus University Medical Centre, Rotterdam, the Netherlands; 2 Department of Public Health, Erasmus University Medical Centre, Rotterdam, the Netherlands; 3 Department of Anaesthesiology, University of Cambridge, UK; 4 Department of Neurosurgery, University Hospital Antwerp, Antwerp, Belgium; 5 Emergency Medicine Research in Sheffield (EMRiS), School of Health and Related Research (ScHARR), Faculty of Medicine, Dentistry and Health, University of Sheffield, Sheffield, UK

**Keywords:** effectiveness, Europe, neurological outcome, prehospital, tracheal intubation, traumatic brain injury

## Abstract

**Background:**

We aimed to study the associations between pre- and in-hospital tracheal intubation and outcomes in traumatic brain injury (TBI), and whether the association varied according to injury severity.

**Methods:**

Data from the international prospective pan-European cohort study, Collaborative European NeuroTrauma Effectiveness Research for TBI (CENTER-TBI), were used (*n*=4509). For prehospital intubation, we excluded self-presenters. For in-hospital intubation, patients whose tracheas were intubated on-scene were excluded. The association between intubation and outcome was analysed with ordinal regression with adjustment for the International Mission for Prognosis and Analysis of Clinical Trials in TBI variables and extracranial injury. We assessed whether the effect of intubation varied by injury severity by testing the added value of an interaction term with likelihood ratio tests.

**Results:**

In the prehospital analysis, 890/3736 (24%) patients had their tracheas intubated at scene. In the in-hospital analysis, 460/2930 (16%) patients had their tracheas intubated in the emergency department. There was no adjusted overall effect on functional outcome of prehospital intubation (odds ratio=1.01; 95% confidence interval, 0.79–1.28; *P*=0.96), and the adjusted overall effect of in-hospital intubation was not significant (odds ratio=0.86; 95% confidence interval, 0.65–1.13; *P*=0.28). However, prehospital intubation was associated with better functional outcome in patients with higher thorax and abdominal Abbreviated Injury Scale scores (*P*=0.009 and *P*=0.02, respectively), whereas in-hospital intubation was associated with better outcome in patients with lower Glasgow Coma Scale scores (*P*=0.01): in-hospital intubation was associated with better functional outcome in patients with Glasgow Coma Scale scores of 10 or lower.

**Conclusion:**

The benefits and harms of tracheal intubation should be carefully evaluated in patients with TBI to optimise benefit. This study suggests that extracranial injury should influence the decision in the prehospital setting, and level of consciousness in the in-hospital setting.

**Clinical trial registration:**

NCT02210221.

**Editor's key points**

• It is difficult to know whether to intubate and institute mechanical ventilatory support for those with traumatic brain injuries.
• This large observational study suggests that the indications for tracheal intubation in the setting of traumatic brain injury should be the extent of extracranial injury and the severity of brain injury.
• Patients with extensive extracranial injury might benefit from intubation before arrival at the hospital.
• Those with impaired level of consciousness as assessed by the Glasgow Coma Scale might benefit from tracheal intubation shortly after they arrive at the hospital.

The burden of traumatic brain injury (TBI) is high: it is a leading cause of injury-related death and disability.^1^ TBI is estimated to be responsible for 287.2 hospital admissions and 11.7 deaths per 100 000 persons per year in Europe.^2^ Mortality rates are higher for moderate and severe TBIs compared with mild TBIs. Although the primary injury arising at the time of impact cannot be mitigated, secondary brain injury arising from subsequent hypoxaemia and hypotension worsens outcome and should be prevented.^3^, ^4^, ^5^

Hypoxaemia and hypotension are both influenced by intubation; tracheal intubation in patients who are not deeply comatose requires induction of anaesthesia and neuromuscular block.^6^^,^^7^ However, injudicious use of anaesthetics and positive pressure ventilation can cause hypotension, particularly in hypovolaemic trauma patients.^8^ Meanwhile, inadequate depth of anaesthesia during laryngoscopy may precipitate hypertension and (further) increase of intracranial pressure (ICP).^9^ Drug-assisted intubation can be technically challenging in patients with TBI, particularly under prehospital conditions. Under these conditions, positioning and lighting may be suboptimal. If there is also associated facial injury present, the risks of a ‘can't intubate can't ventilate’ scenario, or oesophageal intubation, are not negligible. Failure to rapidly control the airway owing to delayed or unsuccessful intubation attempts may lead to, or worsen, hypoxia or hypercapnia. These secondary insults are associated with worse outcomes for TBI patients, and may be mitigated or contributed to by decisions to intubate.^4^^,^^10^, ^11^, ^12^, ^13^

The international guidelines of the Brain Trauma Foundation on intubation in TBI^14^ recommend intubation for patients with more severe injuries. However, the body of evidence underlying this recommendation consists of only class III evidence, mostly from small retrospective studies. The exception is a randomised trial by Bernard and colleagues^15^ showing benefit of prehospital *vs* in-hospital intubation in injured prehospital patients with a Glasgow Coma Scale (GCS) score ≤9. These data have driven recommendations and practice: more severely injured patients, typically with a GCS score of 8 or lower, are intubated more often.^16^ However, the primarily observational associations that underpin this practice recommendation are prone to ‘confounding by indication’ bias.

Possibly partly as a result of the low quality of evidence, guideline adherence varies.^17^ For prehospital intubation (PHI), the estimate lies about 80% adherence, but a large range of 44%–92% adherence is observed in the literature.^18^^,^^19^ There is a need for prospective evidence, sufficiently adjusting for confounding bias.

The aim of this prospective study was to improve evidence supporting the guideline recommendations regarding PHI and in-hospital intubation (IHI). Given the practice variation in intubation, we wanted to assess the effect of intubation both at the patient level and at the trauma system level. In addition, given the guideline recommendations to intubate more severely injured patients, we explored whether GCS score and extracranial injury influence the effect of intubation on functional outcome. Finally, we wanted to replicate the RCT by Bernard and colleagues^15^ in the European setting, by comparing outcome of PHI *vs* intubation at the emergency department (ED) in patients whose tracheas were intubated.

## Methods

This study was reported according to STROBE (Strengthening The Reporting of OBservational Studies in Epidemiology) guidelines.^20^

### Study population

We studied patients who were included in the European, prospective, longitudinal cohort study, Collaborative European NeuroTrauma Effectiveness Research for Traumatic Brain Injury (CENTER-TBI). In this study, data from 4509 all-severity TBI patients in 59 centres throughout Europe had been collected in the period of 2014–2018 and were available for analysis. Further details of the CENTER-TBI study, including rationale for sample size, have been published elsewhere.^21^^,^^22^ A predetermined analysis plan was approved by the management committee before the actual analysis started.

### Patient selection

We excluded patients in whom intubation could not have been considered. For PHI, we therefore excluded patients who arrived to the study hospital without activating emergency medical services (self-presenters). For the IHI analysis, we excluded patients whose tracheas were already intubated on scene.

### Definitions

PHI was defined as intubation at the scene of injury. IHI was defined as intubation at the ED of the study hospital, or intubation at the referring hospital if the patient was transferred. Intubation could include intubation with and without sedation. The best prehospital GCS score was used for the analysis of PHI and for the analysis of PHI *vs* IHI. The GCS score at ED arrival was used for the analysis of IHI. The baseline GCS score was defined as the last GCS score in the ED (after stabilisation). If this was missing, or when the patient was sedated or when the patient's trachea was intubated, a previous measurement moment was used: at ED arrival or prehospital, respectively. Outcome was measured using the Glasgow Outcome Scale – Extended (GOS-E) at 6 months after injury, GOS-E is an eight-point scale that measures functional outcome after TBI.^23^

For risk adjustment, we used variables from the IMPACT (International Mission for Prognosis and Analysis of Clinical Trials in TBI) model^24^ including age, GCS score, pupil reactivity, imaging characteristics (traumatic subarachnoid haemorrhage, epidural haematoma, Marshall CT class), physiological parameters at ED arrival (heart rate, systolic blood pressure, oxygen saturation), and also secondary insults during the ER treatment (hypoxia or hypotension at the ED). Hypoxia was defined as a documented *P*ao_2_ below 8 kPa (60 mm Hg), a documented *S*ao_2_ below 90%, or both, or in case of clinical suspicion (e.g. cyanosis, apnoea, or respiratory distress) when not documented. Hypotension was defined as a documented systolic blood pressure below 90 mm Hg, or in case of clinical suspicion (e.g. shock or absent brachial pulse) when not documented. Moreover, because extracranial injury is also described as a confounder,^25^ we also included abbreviated injury severity (AIS) scores of head, spine/chest, abdominal (including pelvis), limbs, and face. Finally, as literature suggests differences in outcome between men and women,^26^ we assumed sex to be a potential confounder as well.

### Statistical analysis

For the patient-level descriptive analysis, baseline characteristics were compared between the PHI, IHI, and not-intubated (NI) group. Medians and inter-quartile ranges (IQRs) are reported for non-normally distributed variables; for normally distributed variables, means and standard deviations are reported.

Missing data were multiply imputed for the main analyses using the ‘mice’ package.^27^ The missing pattern was assumed to be missing at random. Together with the potential confounders and intubation, GOS-E was included in the imputation model. Five imputed datasets were obtained.

To assess the effect of intubation on outcome, proportional odds logistic regression was performed using intubation as independent variable and GOS-E as dependent variable, with adjustment for confounders. We allowed for non-linear effects by using restricted cubic splines with three degrees of freedom for heart rate, systolic blood pressure, saturation, and age, and with second-degree polynomials for AIS scores. Finally, to assess whether GCS score, abdominal AIS, or thorax AIS influenced the effect of intubation, interaction terms between these characteristics and intubation were added in a consecutive model. We present the effect of intubation as odds ratios (ORs) for more unfavourable outcome and 95% confidence intervals (CIs). The exception is the presentation of the interaction effect: because the interaction effect is based on the combination of two coefficients (the main effect of intubation and the interaction with injury severity), the interpretation is more complex. Instead, we only present the *P*-value of the overall test (likelihood ratio test) for interaction.

To investigate the relationship between intubation practice and outcome at the hospital level, we calculated the adjusted probabilities of intubation based on a multinomial mixed effects regression model. The covariates included in the model were based on previous work,^28^ and include age, GCS score, anatomical injury scales (head/neck thorax/chest, face, and abdomen), and pupil reactivity. A random intercept for centre, conditional on country, was used to adjust for random variation. Because we used multinomial regression, separate random intercepts for each centre were estimated for both outcomes (PHI and IHI). To define the outcome per centre, we calculated mean GOS-E scores per centre. The association between intubation preference and outcome was estimated with linear regression with the random intercepts per centre for IHI and PHI, and IHI or PHI itself as an independent variable and mean GOS-E per centre as a dependent variable. An interaction term between the intubation preference and PHI or IHI was included. The coefficient of the model was divided by 10 to calculate the coefficient per 10% increase in adjusted intubation rate. The coefficient for interaction between preference and intubation was added to the main effect. Only centres with more than 20 included patients were included in this analysis.

## Results

**Fig 1:**
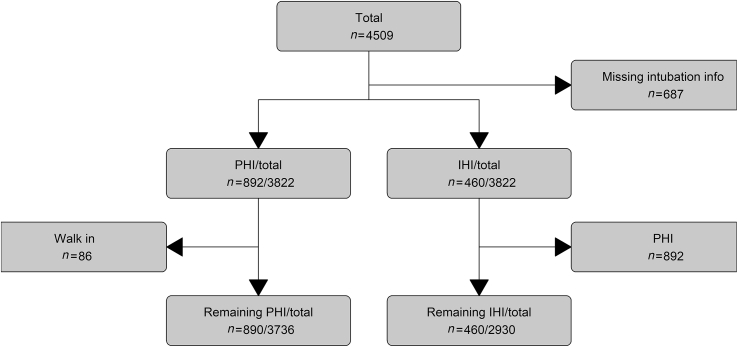
Flowchart showing the number of patients excluded with each criterion. IHI, in-hospital intubation; PHI, prehospital intubation.

The CENTER-TBI database consists of 4509 patients, included across 59 centres in Europe. Information about intubation was present in a total of 3822 (85%) patients, who came from all participating centres (Fig. 1).

### Prehospital intubation

In the PHI analysis, after excluding patients who self-presented at the ED (*n*=86), 3736 patients were included. Of these patients, 890 (24%) underwent tracheal intubation on scene. Of 3166 (85%) patients, a GOS-E was obtained at 6 months follow-up.

**Fig 2:**
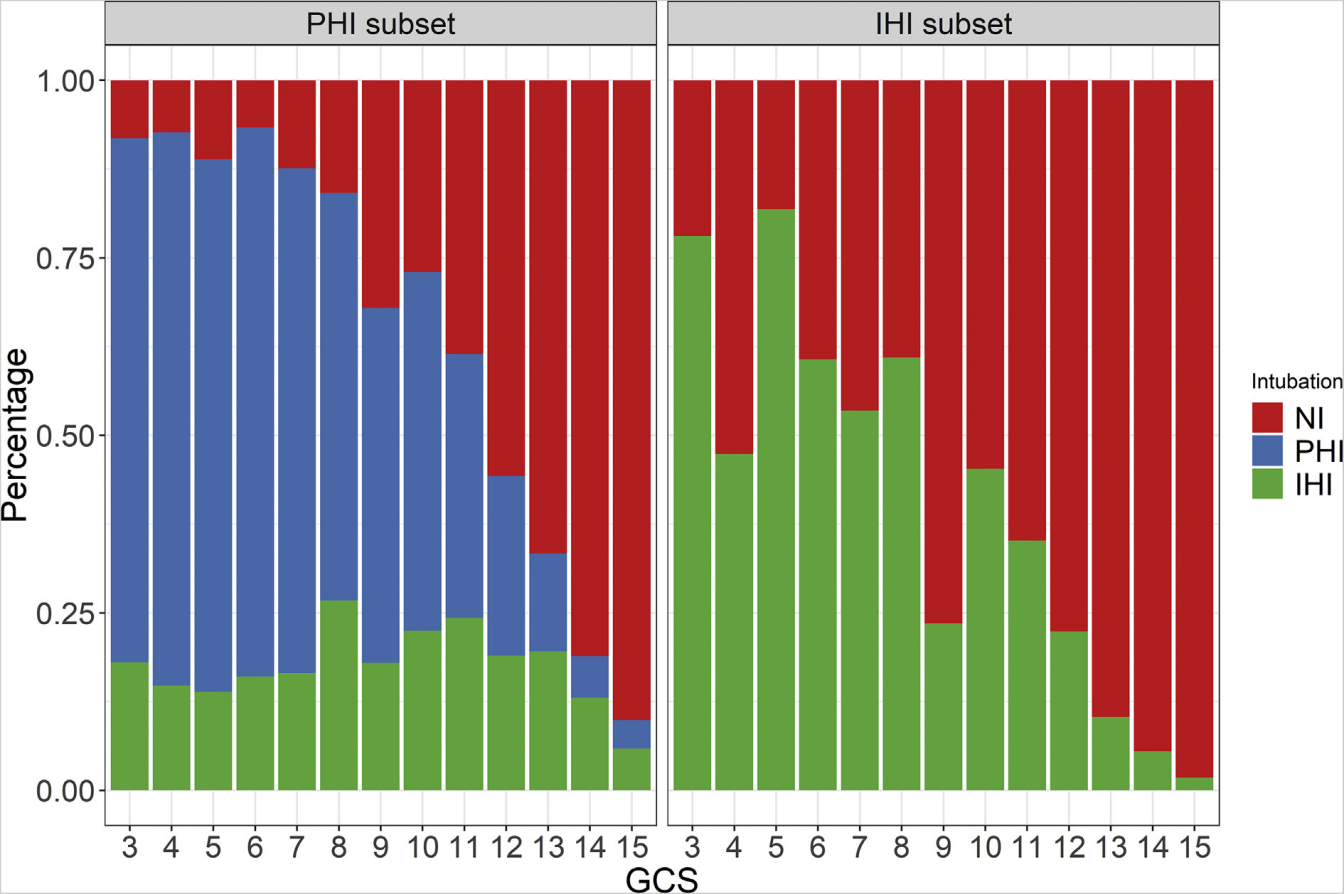
Proportion of non-intubated (NI), prehospitally intubated (PHI) and in-hospital intubated (IHI) patients with a certain Glasgow Coma Scale (GCS) score.

In this PHI subset, 571 (72.4%) of the patients with a prehospital GCS score of 8 or lower had their tracheas intubated on scene, and 212 (12%) of the patients with a prehospital GCS score higher than 8 had their tracheas intubated on scene (Fig. 2). On average, patients that had their tracheas intubated had lower baseline GCS score, were younger, and more often male. Furthermore, based on a threshold AIS > 3, patients who were intubated had a higher proportion of head and cervical spine injury, major chest/spine injury, and abdominal injury. In addition, patients whose tracheas were intubated had more intracranial pathologies, and suffered from more secondary hypoxic and hypotensive insults in the ED (Table 1). These differences were smaller when patients with GCS scores above 8 were excluded (Supplementary Table S1). The hospital stay of patients that required PHI was characterised by a longer total length of stay, and a longer ICU stay, and more days of mechanical ventilation and sedation. In addition, pneumonia was observed more often in these patients, and more extracranial and intracranial surgeries, including decompressive craniectomies. Although the absolute ICP values in patients in whom it was measured did not differ substantially on average, the therapy intensity that they received was higher in patients who required intubation. Finally, the blood glucose concentrations were higher in patients who required intubation, both at day 1 as during the entire stay.

**Table 1:** Baseline table of characteristics of the studied cohort. Regional AIS score >2. ASA, American Society of Anestehsiologists; NI, not intubated; PI, prehospital intubation; IHI, in-hospital intubation; ISS, injury severity score; RTA, road traffic accident; GCS, Glasgow Coma Scale; mGCS, Glasgow Coma Scale, motor component; ED, emergency department; IQR, inter-quartile range; EDH, epidural haematoma; TSAH, traumatic subarachnoid haemorrhage; MLS, midline shift.

	PHI (*n*=890)	NI – PHI subset (*n*=2846)	Missing (%)	*P*-value	IHI (*n*=460)	NI – IHI subset (*n*=2470)	Missing (%)	*P*-value
Age (median [IQR])	44 [25, 60]	52 [33, 68]	0	<0.001	52 [31, 67]	53 [33, 68]	0	0.131
Male (%)	657 (73.8)	1895 (66.6)	0	<0.001	334 (72.6)	1608 (65.1)	0	0.002
Pre-injury ASA physical status			2.6	<0.001			1.7	0.001
1	545 (64.8)	1540 (55.1)			215 (48.8)	1368 (56.1)		
2	227 (27.0)	942 (33.7)			167 (37.9)	803 (32.9)		
3	68 (8.1)	291 (10.4)			49 (11.1)	251 (10.3)		
4	1 (0.1)	24 (0.9)			10 (2.3)	16 (0.7)		
Smoked any time before injury	273 (44.6)	979 (41.7)	20.7	0.204	157 (50.0)	851 (40.3)	17.2	0.001
Drank alcohol any time before injury	189 (31.3)	809 (34.8)	21.7	0.119	112 (36.5)	720 (34.4)	18.1	0.518
Major∗ head injury (%)	*851 (95.6)*	*1960 (68.9)*	0	<0.001	441 (95.9)	1569 (63.5)	0	<0.001
Major∗ chest/spine injury (%)	*408 (45.8)*	*436 (15.3)*	0	<0.001	135 (29.3)	303 (12.3)	0	<0.001
Major∗ face injury (%)	261 (29.3)	341 (12.0)	0	<0.001	106 (23.0)	237 (9.6)	0	<0.001
Major∗ abdominal injury (%)	139 (15.6)	148 (5.2)	0	<0.001	40 (8.7)	108 (4.4)	0	<0.001
Major∗ external injury (%)	40 (4.5)	45 (1.6)	0	<0.001	12 (2.6)	33 (1.3)	0	0.067
Major∗ extremity injury (%)	235 (26.4)	356 (12.5)	0	<0.001	80 (17.4)	277 (11.2)	0	<0.001
Cause (%)			2	<0.001			2	0.105
RTA	482 (55.5)	1059 (38.1)			173 (39.8)	903 (37.2)		
Fall	284 (32.7)	1306 (47.0)			184 (42.3)	1165 (48.0)		
Other	59 (6.8)	230 (8.3)			41 (9.4)	203 (8.4)		
Violence/suicide	44 (5.1)	186 (6.7)			37 (8.5)	155 (6.4)		
GCS score baseline (median [IQR])	4 [3, 8]	15 [13, 15]	2	<0.001	8 [5, 13]	15 [14, 15]	2	<0.001
GCS score prehospital (median [IQR])	6 [3, 9]	14 [13, 15]	36	<0.001	10 [6, 14]	15 [14, 15]	40	<0.001
GCS score at ED arrival (median [IQR])	3 [3, 3]	15 [14, 15]	17	<0.001	8 [5, 12]	15 [14, 15]	12	<0.001
mGCS score baseline (median [IQR])	1 [1, 4]	6 [6, 6]	1	<0.001	5 [1, 6]	6 [6, 6]	1	<0.001
mGCS score prehospital (median [IQR])	3 [1, 5]	6 [6, 6]	36	<0.001	5 [3, 6]	6 [6, 6]	40	<0.001
mGCS score at ED arrival (median [IQR])	1 [1, 1]	6 [6, 6]	16	<0.001	5 [1, 6]	6 [6, 6]	12	<0.001
Unreactive pupils, baseline (%)			4	<0.001			5	<0.001
0	592 (69.6)	2578 (94.7)			355 (81.1)	2293 (97.2)		
1	71 (8.4)	71 (2.6)			33 (7.5)	40 (1.7)		
2	187 (22.0)	74 (2.7)			50 (11.4)	26 (1.1)		
Heart rate at ED arrival, mean (sd)	89 (24)	83 (18)	8	<0.001	84 (21)	82 (17)	8	0.184
SBP at ED arrival, mean (sd)	129 (31)	141 (26)	7	<0.001	140 (32)	141 (25)	7	0.834
*S*po_2_ at ED arrival, median [IQR]	100 [98, 100]	98 [96, 100]	12	<0.001	98 [96, 100]	98 [97, 100]	12	0.820
Hypoxia at ED (%)	175 (20.6)	105 (3.9)	4	<0.001	62 (14.9)	45 (1.9)	4	<0.001
Hypotension at ED (%)	189 (22.2)	94 (3.4)	3	<0.001	44 (10.4)	51 (2.1)	3	<0.001
EDH (%)	133 (16.1)	253 (9.6)	7	<0.001	78 (20.0)	182 (7.8)	6	<0.001
TSAH (%)	606 (73.2)	1039 (39.3)	7	<0.001	276 (70.8)	779 (33.5)	6	<0.001
Marshall CT class (%)			10	<0.001			9	<0.001
No visible pathology on CT	77 (9.7)	1143 (44.6)			35 (9.4)	1151 (50.9)		
Cisterns present, MLS <5 mm	390 (48.9)	968 (37.8)			135 (36.1)	850 (37.6)		
Cisterns compressed or absent	110 (13.8)	74 (2.9)			31 (8.3)	43 (1.9)		
Mass lesion	220 (27.6)	376 (14.7)			173 (46.3)	217 (9.6)		
Arrival time (min)	20 [11, 30]	15 [10, 27]	44	<0.001	14 [8, 24]	15 [10, 27]	44	0.020
On-scene time (min)	35 [25, 51]	20 [14, 30]	48	<0.001	23 [15, 32]	20 [14, 30]	49	0.009
Travel time (min)	20 [12, 35]	16 [10, 25]	48	<0.001	13 [9, 22]	16 [10, 25]	49	0.002

**Fig 3:**
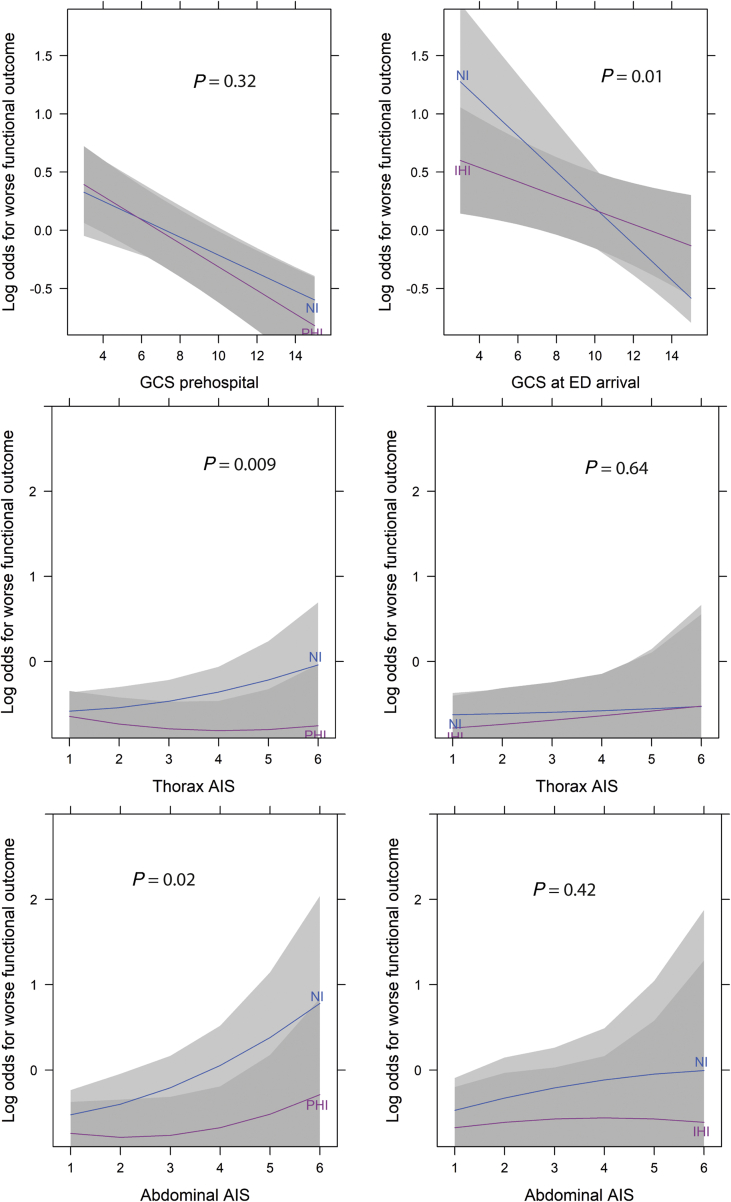
Treatment effect estimates on functional outcome, allowing for interaction of intubation with GCS score, head AIS, and abdominal AIS. The left panel shows the results for prehospital intubation (PHI), and the right for in-hospital intubation (IHI). The effect is displayed for the statistically average patient, with the median (continuous) or mode (categorical) for all other characteristics. AIS, abbreviated injury severity; GCS, Glasgow Coma Scale.

Before adjusting for possible confounders, PHI was associated with worse functional outcome (OR=6.70; 95% CI, 5.75–7.81; *P*<0.001). After adjustment, there was no evidence of an effect of PHI on functional outcome (OR=1.01; 95% CI, 0.79–1.28; *P*=0.96; Table 2). The interaction with prehospital GCS score was not significant (*P*=0.32), but the effect with extracranial injury was significant: PHI was associated with better functional outcome in patients with higher thorax and abdominal AIS scores (*P*=0.009 for thorax AIS and *P*=0.02 for abdominal AIS; Fig. 3).

**Table 2:** Effect of prehospital (PHI) and in-hospital intubation (IHI) on lower functional outcome (GOS-E). An odds ratio greater than 1 indicates a higher probability of lower functional outcome (harmful). ∗For age, sex, baseline GCS, pupil reactivity, heart rate/systolic blood pressure/saturation at arrival, AIS scores of head/spine/abdominal/face regions, traumatic subarachnoid haemorrhage, epidural haematoma, CT class, hypoxia/hypotension at the emergency department. ^†^Only in patients with GCS ≤9, who received intubation. GCS, Glasgow Outcome Scale; GOS-E, Glasgow Outcome Scale – Extended.

Intubation	Unadjusted	Adjusted∗
PHI	6.70 (5.75–7.81)	1.01 (0.79–1.28)
IHI	6.13 (5.05–7.44)	0.86 (0.65–1.13)
PHI *vs* IHI^†^	0.87 (0.66–1.15)	0.90 (0.65–1.23)

### In-hospital intubation

In the in-hospital analysis, after excluding patients whose tracheas were intubated on scene, 2930 patients were included (Fig. 1). Of these patients, 460 (16%) patients had their tracheas intubated at the ED. Of 2458 (84%) patients, a GOS-E was obtained at 6 months follow-up.

In this IHI subset, 140 (65%) of the patients with a GCS score of 8 or lower at ED arrival had their tracheas intubated at the ED (41 [46%] of these had GOS-E scores ≤4 at 6 months), and 127 (6%) of the patients with a GCS score higher than 8 at ED arrival. On average, they had lower baseline GCS score (Fig. 2). In addition, they were more often male, had a higher proportion of major head injury, and a higher proportion of major extracranial injury. Moreover, patients who had their tracheas intubated had more intracranial pathologies and suffered from more secondary insults (Table 1). These differences were smaller when patients with GCS scores above 8 were excluded (Supplementary Table S1). The hospital stay of patients that required IHI was characterised by a longer total length of stay, and a longer ICU stay, and more days of mechanical ventilation and sedation. In addition, pneumonia was observed more often in these patients, and more extracranial and intracranial surgeries, including decompressive craniectomies. Although the absolute ICP value in patients in whom it was measured did not differ substantially on average, the therapy intensity that they received was higher in patients who required intubation. Finally, the blood glucose concentrations were higher in patients who required intubation, both at day 1 as during the entire stay.

Before adjusting for confounders, IHI was associated with worse functional outcome (OR=6.13; 95% CI, 5.05–7.44; *P*<0.001). After adjustment, there was no conclusive evidence of an effect of IHI functional outcome (OR=0.86; 95% CI, 0.65–1.13; *P*=0.28; Table 2). The interaction with extracranial injury was not significant, but the effect with GCS score was significant (*P*=0.01): IHI was associated with better functional outcome in patients with GCS scores of 10 or lower at ED arrival (Fig. 3).

### Prehospital *vs* in-hospital intubation

Compared with patients whose tracheas were intubated at the ED, patients with a GCS score ≤9 whose tracheas were intubated on scene were younger, had more extracranial injuries, had lower prehospital GCS scores, had more unreactive pupils, and suffered more from secondary insults. Moreover, the median arrival time was 18 min (IQR, 10–29), the median on-scene time was 30 min (IQR, 20–45), and the median travel time to the hospital was 18 min (IQR, 11–30; Table 1). The crude and adjusted effect of PHI *vs* IHI was beneficial, but not significant: the crude OR for lower GOS-E was 0.87 (95% CI, 0.66–1.15), and the adjusted OR for a lower GOS-E was 0.90 (95% CI, 0.65–1.23). The interaction with injury severity (both GCS score and extracranial injury), was not significant.

### Intubation practice

**Fig 4:**
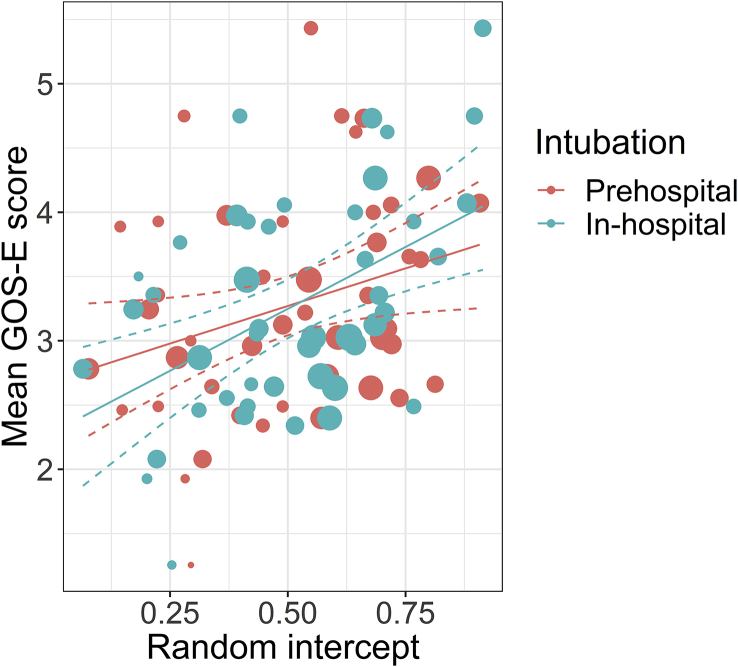
Outcome with centres with different frequencies of intubation. On the *x*-axis, the values of the random intercept values of the mixed-effects multinomial model are displayed. These can be interpreted as the adjusted intubation rate (the higher the value, the higher the intubation rate). On the *y*-axis, the mean Glasgow Outcome Scale – Extended (GOS-E) for the patients in that centre is displayed. Both prehospital and in-hospital intubation are shown. The sizes of the dots represent the sample size of the centres (corresponding to the inverse variance). The mean and 95% confidence interval (CI) is displayed in black.

The intubation rates ranged from 0% to 60% for PHI, and from 2% to 56% for IHI (Supplementary Fig. S1). Higher adjusted intubation rates per hospital were associated with higher mean GOS-E scores (Fig. 4). The relationship was not significantly different for PHI or IHI (*P*=0.34): for every 10% increase in PHI rate, the mean GOS-E increased with 0.12 (95% CI, 0.01–0.22; *P*=0.04), whereas for every 10% increase in IHI rate, the mean GOS-E increased with 0.19 (95% CI, 0.08–0.30; *P*=0.03).

## Discussion

This study aimed to provide insight into the effect of intubation on outcome in TBI patients. We performed a patient-level analysis, which is complicated because patients whose tracheas were intubated had sustained more severe trauma. After adjustment for possible confounders, there was no evidence for an overall effect of intubation on functional outcome in TBI patients. Although higher or lower GCS scores did not influence the effect of intubation in the prehospital setting, intubation at the ED seemed to have a more beneficial effect in patients with lower GCS scores. In contrast, higher extracranial injury AIS scores mainly influenced the effect of intubation in the prehospital setting, where intubation was associated with better functional outcome in patients with higher extracranial injury AIS scores. The findings of the RCT by Bernard and colleagues^15^ were not reinforced by our results: PHI was not associated with better functional outcome than IHI. Finally, higher adjusted intubation rates per centre were associated with better functional outcomes.

At the patient level, previous observational studies that assessed the effect of intubation on outcome primarily counterintuitively showed a harm of intubation.^29^ Observational studies are inherently prone to confounding bias. In an attempt to adjust for this bias, some recent studies used propensity score matching.^30^^,^^31^ These studies also showed an association of intubation with unwanted outcomes in severe TBI patients: these studies found worsened admission oxygenation and even higher mortality. A postintubation surge in ICP or occurrence of hypotension could increase mortality. However, interpreting this relationship as causal is not appropriate, because the purpose of intubation is to secure oxygenation. Rather, these studies are more likely to suffer from residual confounding bias. Our study extensively corrected for potential confounders, which resulted in a large apparent change in the effect of intubation before and after adjustment. Although the effect of intubation was not statistically significant overall, the effect of intubation, especially at the ED, appeared more likely to be beneficial than harmful. This is in accordance with a study by Davis and colleagues.^25^ This study found a small positive effect of intubation when adjusted for Trauma Score and Injury Severity Score (TRISS). This effect was particularly found in patients who would otherwise be expected to die: those with a very high TRISS score. The finding of a more beneficial effect for more severely injured patients is in accordance with our finding that the benefit of intubation is higher in patients with lower GCS scores and higher extracranial AIS scores. Although this was previously assumed from a physiological perspective,^6^ it has not been confirmed empirically extensively.

In TBI, particularly in patients with more severe TBI or with extracranial injury that impacts on respiratory physiology, the benefits of intubation appear to outweigh the harms. The potential harms of intubation are mostly associated with the administration of sedatives. These drugs are known to cause vasodilation and therefore hypotension. The latter is known to be associated with worse outcome.^32^ In addition, patients whose tracheas are intubated are often hyperventilated,^33^ which again worsens outcomes.^34^^,^^35^ However, hypoxia and aspiration, also known to be harmful,^36^^,^^37^ can be prevented through intubation. Our results, together with the data from Davis and colleagues,^25^ suggest that the prevention of hypoxia and aspiration apparently outweighs the harm of both hypotension and hyperventilation in more severe TBI. We found that the severity of both extracranial and intracranial injuries influence the benefit of intubation. Severity of extracranial injury primarily influences intubation in the prehospital setting, whereas in IHI intracranial injury seems more important: intubation was associated with better functional outcome in patients with a GCS score lower than 10. In our study, only a small proportion of patients with a GCS score higher than 8 received tracheal intubation. This is in agreement with current Advanced Trauma Life Support (ATLS) guidelines and prior literature, which recommends intubation in patients with a GCS score of 8 or lower.^6^ However, based on the current study, shifting the ‘intubation threshold’ to a GCS score of 10 or lower (especially at the ED) could be considered.

PHI was not found to be more beneficial than IHI, in contrast to the findings of Bernard and colleagues.^15^ On one hand, it is possible that our results are biased by confounding by indication and hence may not have been able to demonstrate the beneficial effect of PHI. On the other hand, the benefit of PHI demonstrated in an Australian setting by Bernard and colleagues^15^ might not directly be generalisable to Europe. In Europe, the density of hospitals is higher, which probably results in shorter prehospital times: the travel time (time from departure from scene until arrival in a hospital) in particular was 10 min shorter in CENTER-TBI. The advantage of prehospital *vs* IHI is that the airway is secured at an earlier phase. In Europe, the difference in time between a secured airway because of PHI *vs* IHI might be too small to observe a benefit of PHI: the risks of intubating in a less-controlled environment might not be outweighed by the benefits of an earlier secured airway. This hypothesis, however, should be confirmed.

Higher rates of intubation were associated with more favourable outcome. However, this result is not directly applicable to patient-level decision making. Because of ecological bias,^38^ it should rather be explained by differences in resources. These differences in resources contribute to the large variation in intubation rates.^28^ Therefore, this finding should stimulate support in improving current European trauma systems, especially in terms of coverage in appropriate intubation.

A limitation of our study is the observational aspect of our study. In the context of an observational study, it cannot be assumed that confounding bias is entirely corrected for using covariate adjustment. There remains a possibility of unmeasured confounding, which is difficult to overcome. For PHI, in particular, we were not able to adjust for prehospital physiology. Therefore, we recommend future observational studies in this field to meticulously register prehospital physiology, including end-tidal CO_2_. Nevertheless, the estimates for in-hospital and PHI change similarly after adjustment, which supports our conclusion. The lack of details in the prehospital setting drives another limitation, because it complicates the adjustment for GCS score. For PHI, we adjust for the best prehospital GCS score. However, the most appropriate GCS score to account for the effect of intubation is the GCS score before intubation. There might be some subtle differences in adjustment that might have been missed because of that lack of details.

The size and international aspect of our study support generalisability. Our study also suggests a more liberal GCS score threshold should perhaps influence decisions regarding tracheal intubation, especially when considering IHI.

## Conclusions

At the systems level, higher intubation rates are associated with better functional outcome. This finding probably reflects that more resourced trauma systems have better outcomes. This finding warrants support for developing trauma systems throughout Europe.

At the patient level, intubation does not seem to be associated with better or worse outcome in the general TBI population. However, in more severely injured patients, intubation was associated with better functional outcome. Moreover, patients with TBI and significant extracranial injury seemed to benefit most from PHI, whereas the impact of ED intubation was most influenced mostly by GCS score. In addition, in this multicentre study, PHI was not associated with better functional outcome than IHI for patients with TBI.

## Authors’ contributions

Conceptualisation: BYG, MK

Data curation: BYG

Formal analysis: BYG

Investigations: BYG, CAS, DN, DKM, AM, FL, HFL

Methodology: BYG, DN, MK, HFL

Project administration: BYG, MK, HFL

Software acquisition: BYG

Supervision: MK, HFL

Resources: HFL

Funding acquisition: DKM, AM, FL, HFL

Validation of the results: BYG, CAS

Visualisation: BYG, CAS

Writing of the original draft: BYG, CAS, FL, MK, HFL

Review of the manuscript: BYG, CAS, DKM, AM, FL, MK, HFL

Editing of the manuscript: BYG, CAS, DN, DKM, AM, FL, MK, HFL

Revision of the manuscript: BYG, CAS, DN, AM, HFL

## Declarations of interest

The authors declare that they have no conflicts of interest.

## Funding

European Union 7th Framework Program (EC Grant 602150). Additional funding from 10.13039/501100007731Hannelore Kohl Stiftung (Germany), OneMind (USA), and Integra LifeSciences Corporation (USA). The funders had no role in the study design, enrolment, collection of data, writing, or publication decisions.
