# Supplementary material for: Tracheal intubation in traumatic brain injury: a multicentre prospective observational study
Source: Br J Anaesth. 2020 Jul 31;125(4):505–17. doi: 10.1016/j.bja.2020.05.067 (PMC7565908; doi:10.1016/j.bja.2020.05.067)
Supplement: Multimedia component 1 [file mmc1.docx]

## Supplementary material

Supplemental Table 1, baseline table of characteristics of patients with GCS ≤8 (prehospital GCS for the PHI subset, GCS at ED arrival for the IHI subset).

|  | PHI (n=571) | NI – PHI subset (n=218) | p-value | IHI (n=140) | NI – IHI subset (n=75) | p-value |
| --- | --- | --- | --- | --- | --- | --- |
| Age (median [IQR]) | 43 [25, 59] | 47 [30, 61] | 0.063 | 50 [30, 66] | 55 [36, 69] | 0.376 |
| Male (%) | 423 (74.1) | 166 (76.1) | 0.613 | 175 (76.8) | 62 (69.7) | 0.481 |
| Pre-injury ASA class |  |  | <0.001 |  |  | 0.590 |
| 1 | 361 (66.7) | 95 (46.3) |  | 62 (46.3) | 34 (49.3) |  |
| 2 | 135 (25.0) | 79 (38.5) |  | 52 (38.8) | 28 (40.6) |  |
| 3 | 44 ( 8.1) | 27 (13.2) |  | 17 (12.7) | 7 (10.1) |  |
| 4 | 1 ( 0.2) | 4 ( 2.0) |  | 3 ( 2.2) | 0 ( 0.0) |  |
| Smoked any time prior to injury | 164 (43.6) | 68 (48.6) | 0.365 | 48 (53.3) | 21 (47.7) | 0.670 |
| Drank alcohol any time prior to injury | 121 (32.4) | 61 (43.6) | 0.024 | 40 (44.9) | 22 (47.8) | 0.892 |
| Major head injury (%) | 553 (96.8) | 200 (91.7) | 0.004 | 226 (99.1) | 87 (97.8) | 1.000 |
| Major chest/spine injury (%) | 269 (47.1) | 62 (28.4) | <0.001 | 65 (28.5) | 20 (22.5) | 0.378 |
| Major face injury (%) | 175 (30.6) | 38 (17.4) | <0.001 | 52 (22.8) | 10 (11.2) | 0.040 |
| Major abdominal injury (%) | 91 (15.9) | 14 ( 6.4) | 0.001 | 18 ( 7.9) | 4 ( 4.5) | 0.555 |
| Major external injury (%) | 29 ( 5.1) | 8 ( 3.7) | 0.516 | 9 ( 3.9) | 1 ( 1.1) | 1.000 |
| Major extremity injury (%) | 153 (26.8) | 35 (16.1) | 0.002 | 38 (16.7) | 10 (11.2) | 0.888 |
| Cause (%) |  |  | <0.001 |  |  | 0.603 |
| RTI | 317 (56.9) | 69 (33.2) |  | 76 (35.5) | 28 (32.9) |  |
| fall | 170 (30.5) | 108 (51.9) |  | 104 (48.6) | 50 (58.8) |  |
| Other | 38 ( 6.8) | 10 ( 4.8) |  | 17 ( 7.9) | 6 ( 7.1) |  |
| Violence/suicide | 32 ( 5.7) | 21 (10.1) |  | 17 ( 7.9) | 1 ( 1.2) |  |
| GCS baseline (median [IQR]) | 3 [3, 6] | 6 [4, 10] | <0.001 | 5 [3, 7] | 6 [4, 7] | 0.014 |
| GCS prehospital (median [IQR]) | 4 [3, 7] | 5 [3, 7] | 0.025 | 7 [4, 11] | 8 [5, 10] | 0.683 |
| GCS at ED arrival (median [IQR]) | 3 [3, 3] | 7 [4, 10] | <0.001 | 6 [3, 8] | 7 [4, 8] | 0.019 |
| mGCS baseline (median [IQR]) | 1 [1, 3] | 4 [1, 5] | <0.001 | 2 [1, 5] | 3 [1, 5] | 0.094 |
| mGCS prehospital (median [IQR]) | 2 [1, 4] | 2 [1, 4] | 0.343 | 4 [1, 5] | 4 [2, 5] | 0.835 |
| mGCS at ED arrival (median [IQR]) | 1 [1, 1] | 4 [1, 5] | <0.001 | 3 [1, 5] | 4 [1, 5] | 0.125 |
| Unreactive pupils baseline (%) |  |  | <0.001 |  |  | 0.678 |
| 0 | 352 (63.4) | 168 (81.2) |  | 160 (73.4) | 64 (74.4) |  |
| 1 | 57 (10.3) | 15 ( 7.2) |  | 23 (10.6) | 7 ( 8.1) |  |
| 2 | 146 (26.3) | 24 (11.6) |  | 35 (16.1) | 15 (17.4) |  |
| Heart rate at ED arrival (mean (sd)) | 90 (24) | 87 (21) | 0.133 | 82 (21) | 84 (21) | 0.477 |
| SBP at ED arrival (mean (sd)) | 126 (31) | 141 (30) | <0.001 | 142 (32) | 146 (30) | 0.978 |
| SpO2 at ED arrival (median [IQR]) | 100 [97, 100] | 99 [96, 100] | 0.004 | 99 [96, 100] | 99 [97, 100] | 0.348 |
| Hypoxia at ED (%) | 131 (24.0) | 36 (17.6) | 0.073 | 40 (19.7) | 7 ( 8.6) | 0.014 |
| Hypotension at ED (%) | 137 (25.0) | 16 ( 7.8) | <0.001 | 21 (10.2) | 4 ( 5.1) | 0.275 |
| EDH (%) | 79 (14.8) | 23 (12.4) | 0.476 | 28 (14.5) | 11 (14.1) | 1.000 |
| TSAH (%) | 399 (75.0) | 132 (71.0) | 0.326 | 146 (75.6) | 49 (62.8) | 0.145 |
| Marshall CT class (%) |  |  | 0.006 |  |  | 0.715 |
| No visible pathology on CT | 43 ( 8.4) | 25 (14.0) |  | 14 ( 7.6) | 5 ( 6.9) |  |
| Cisterns present, MLS < 5 mm | 231 (45.0) | 61 (34.3) |  | 52 (28.3) | 18 (25.0) |  |
| Cisterns compressed or absent | 80 (15.6) | 21 (11.8) |  | 19 (10.3) | 12 (16.7) |  |
| Mass lesion | 159 (31.0) | 71 (39.9) |  | 99 (53.8) | 37 (51.4) |  |
| Arrival time, min | 20 [10, 30] | 14 [7, 23] | <0.001 | 14 [8, 24] | 15 [10, 25] | 0.818 |
| On-scene time, min | 34 [24, 50] | 20 [14, 31] | <0.001 | 22 [15, 32] | 20 [13, 30] | 0.186 |
| Travel time, min | 20 [12, 34] | 13 [7, 21] | <0.001 | 15 [8, 22] | 17 [10, 22] | 0.884 |
| NI = not intubated; PI = prehospitally intubated; IHI = in-hospitally intubated; ISS = injury severity score; RTA = road traffic accident; GCS = Glasgow coma scale; mGCS = Glasgow coma scale, motor component; ED = emergency department; IQR = interquartile range; EDH = epidural hematoma; TSAH = traumatic subarachnoid hemorrhage; MLS = midline shift; CT = computed tomography. | | | | | | |


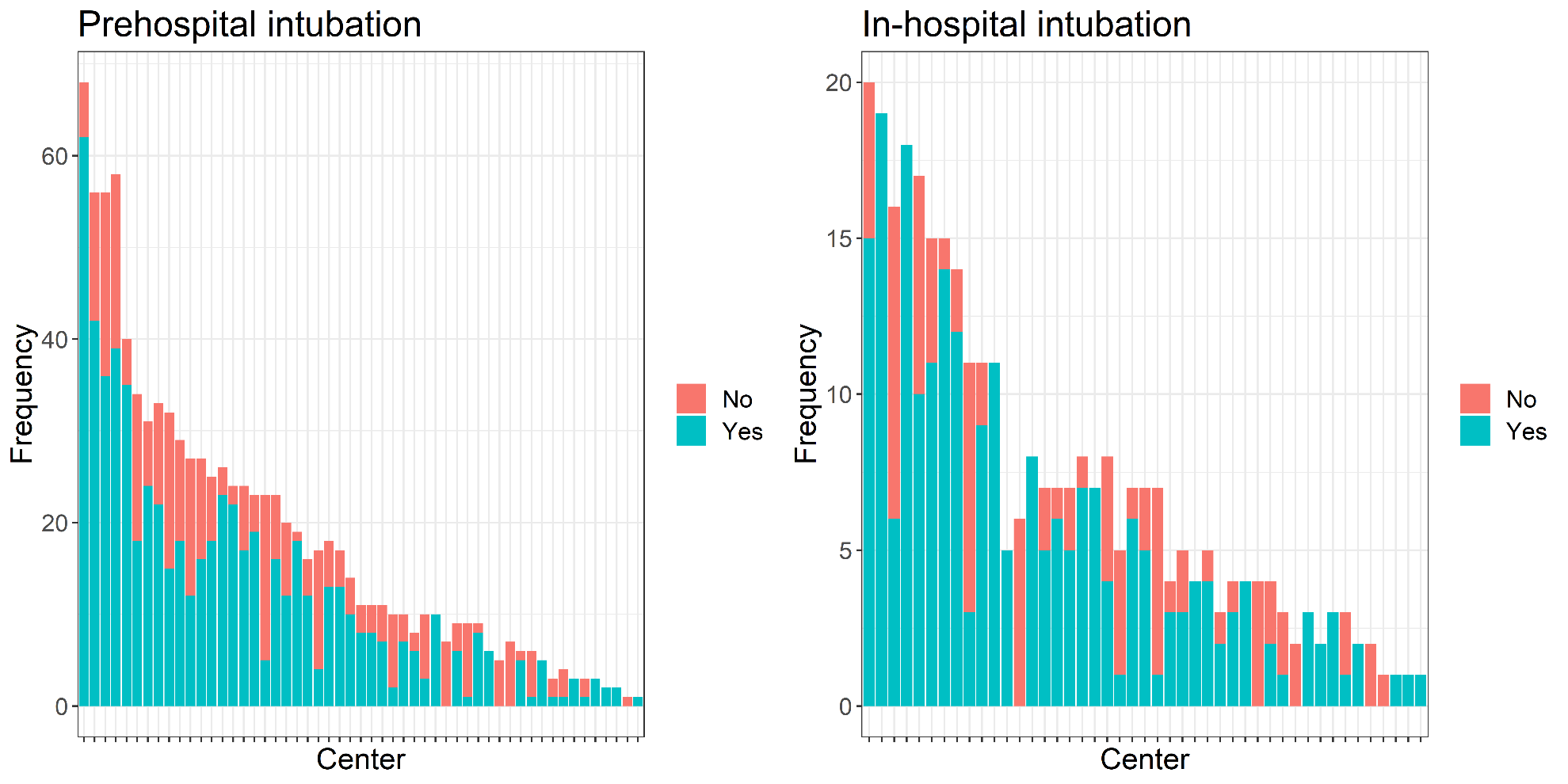


Supplemental Figure 1, bar chart showing the intubation rates per hospital in patients with GCS ≤ 8. The centers are anonymized.

Supplemental table 2, in-hospital clinical course.

|  | PHI (n=890) | NI – PHI subset (n=2846) | Missing (%) | P-value | IHI (n=460) | NI – IHI subset (n=2470) | Missing (%) | P-value |
| --- | --- | --- | --- | --- | --- | --- | --- | --- |
| LOS ICU (median [IQR]) | 11.1 [3.3, 19.9] | 3.9 [1.6, 10.9] | 48.8 | <0.001 | 8.0 [3.4, 16.5] | 2.5 [1.2, 7.0] | 62.5 | <0.001 |
| LOS total (median [IQR]) | 18.8 [7.0, 35.8] | 3.7 [1.0, 11.1] | 2.3 | <0.001 | 16.0 [7.6, 32.2] | 2.6 [0.8, 7.8] | 1.8 | <0.001 |
| Days sedated (median [IQR]) | 5.0 [2.0, 8.0] | 1.0 [0.0, 6.0] | 68.0 | <0.001 | 4.0 [2.0, 8.0] | 0.0 [0.0, 2.0] | 77.8 | <0.001 |
| Days mechanically ventilated (median [IQR]) | 7.5 [2.0, 15.0] | 3.0 [1.0, 10.0] | 60.6 | <0.001 | 5.0 [2.0, 12.0] | 1.0 [0.0, 7.0] | 75.1 | <0.001 |
| Median SBP first day (mean (SD)) | 127.0 (18.7) | 129.5 (18.1) | 68.5 | 0.021 | 128.6 (17.4) | 130.4 (18.9) | 79.3 | 0.222 |
| Median of median SBP per day (mean (SD)) | 131.3 (15.4) | 133.0 (15.9) | 76.1 | 0.116 | 132.4 (15.0) | 133.3 (16.7) | 84.1 | 0.571 |
| Median DBP first day (mean (SD)) | 61.8 (10.3) | 63.2 (10.5) | 68.6 | 0.020 | 61.1 (9.2) | 65.2 (11.3) | 79.3 | <0.001 |
| Median of median DBP per day (mean (SD)) | 62.1 (8.4) | 64.1 (9.6) | 76.2 | 0.001 | 61.6 (9.1) | 66.0 (9.7) | 84.2 | <0.001 |
| Median ICP first day (median [IQR]) | 11.0 [7.0, 15.0] | 11.0 [7.0, 15.5] | 80.7 | 0.891 | 11.0 [7.0, 16.0] | 10.8 [7.0, 14.6] | 89.4 | 0.566 |
| Median of median DBP per day (median [IQR]) | 11.0 [8.5, 15.0] | 12.0 [9.0, 16.0] | 90.7 | 0.164 | 11.5 [8.6, 16.0] | 13.0 [10.4, 16.1] | 94.9 | 0.200 |
| Glucose first day (median [IQR]) | 7.4 [6.3, 9.2] | 6.8 [5.9, 8.2] | 17.7 | <0.001 | 7.4 [6.3, 8.8] | 6.7 [5.8, 8.0] | 21.9 | <0.001 |
| Median glucose per day (median [IQR]) | 7.3 [6.5, 8.3] | 6.8 [5.9, 7.9] | 17.7 | <0.001 | 7.3 [6.5, 8.2] | 6.6 [5.8, 7.8] | 21.9 | <0.001 |
| Highest saturation first day (median [IQR]) | 99.3 [98.6, 100.0] | 99.1 [98.2, 100.0] | 57.6 | 0.029 | 99.1 [98.6, 99.9] | 99.0 [98.0, 100.0] | 71.4 | 0.299 |
| Median daily highest saturation (median [IQR]) | 99.0 [98.2, 99.6] | 98.9 [98.0, 99.6] | 57.6 | 0.078 | 98.9 [98.2, 99.4] | 99.0 [97.8, 100.0] | 71.4 | 0.880 |
| Pneumonia (%) | 159 (18.6) | 104 ( 9.4) | 47.5 | <0.001 | 71 (15.9) | 35 ( 5.2) | 61.8 | <0.001 |
| Cranial surgery (%) | 344 (39.0) | 443 (19.1) | 14.3 | <0.001 | 234 (51.4) | 223 (11.6) | 18.9 | <0.001 |
| Decompressive craniectomy (%) | 144 (16.3) | 128 ( 5.5) | 14.3 | <0.001 | 84 (18.5) | 45 ( 2.3) | 18.9 | <0.001 |
| Extracranial surgery (%) | 312 (35.4) | 370 (15.9) | 14.3 | <0.001 | 112 (24.6) | 260 (13.5) | 18.9 | <0.001 |
| Cumulative TIL, median [IQR] | 25.0 [8.0, 57.0] | 5.0 [0.0, 27.0] | 47.7 | <0.001 | 21.0 [6.0, 51.0] | 2.0 [0.0, 11.0] | 62.5 | <0.001 |
| NI = not intubated; PI = prehospitally intubated; IHI = in-hospitally intubated; LOS = length of stay; ICU = intensive care unit; SBP = Systolic blood pressure; DBP = diastolic blood pressure; ICP = intracranial pressure; TIL = therapy intensity level | | | | | | | | |
